# Development and validation of a machine learning model to predict imminent new vertebral fractures after vertebral augmentation

**DOI:** 10.1186/s12891-023-06557-w

**Published:** 2023-06-09

**Authors:** Yang Jiang, Jinhui Cai, Yurong Zeng, Haoyi Ye, Tingqian Yang, Zhifeng Liu, Qingyu Liu

**Affiliations:** 1 Department of Radiology, The Seventh Affiliated Hospital, Sun Yat-Sen University, Shenzhen, China; 2 Department of Radiology, Huizhou Central People’s Hospital, Huizhou, China; 3 Department of Radiology, The Fourth Affiliated Hospital of Guangzhou Medical University, Guangzhou, China

**Keywords:** Osteoporosis, Vertebral fracture, Vertebral augmentation, Radiomics, Machine learning

## Abstract

**Background:**

Accurately predicting the occurrence of imminent new vertebral fractures (NVFs) in patients with osteoporotic vertebral compression fractures (OVCFs) undergoing vertebral augmentation (VA) is challenging with yet no effective approach. This study aim to examine a machine learning model based on radiomics signature and clinical factors in predicting imminent new vertebral fractures after vertebral augmentation.

**Methods:**

A total of 235 eligible patients with OVCFs who underwent VA procedures were recruited from two independent institutions and categorized into three groups, including training set (*n* = 138), internal validation set (*n* = 59), and external validation set (*n* = 38). In the training set, radiomics features were computationally retrieved from L1 or adjacent vertebral body (T12 or L2) on T1-w MRI images, and a radiomics signature was constructed using the least absolute shrinkage and selection operator algorithm (LASSO). Predictive radiomics signature and clinical factors were fitted into two final prediction models using the random survival forest (RSF) algorithm or COX proportional hazard (CPH) analysis. Independent internal and external validation sets were used to validate the prediction models.

**Results:**

The two prediction models were integrated with radiomics signature and intravertebral cleft (IVC). The RSF model with C-indices of 0.763, 0.773, and 0.731 and time-dependent AUC (2 years) of 0.855, 0.907, and 0.839 (*p* < 0.001 for all) was found to be better predictive than the CPH model in training, internal and external validation sets. The RSF model provided better calibration, larger net benefits (determined by decision curve analysis), and lower prediction error (time-dependent brier score of 0.156, 0.151, and 0.146, respectively) than the CPH model.

**Conclusions:**

The integrated RSF model showed the potential to predict imminent NVFs following vertebral augmentation, which will aid in postoperative follow-up and treatment.

**Supplementary Information:**

The online version contains supplementary material available at 10.1186/s12891-023-06557-w.

## Background

Fractures due to osteoporosis are becoming more common in women over 55 years and men over 65 years, resulting in significant bone-related morbidities, increased mortality, and a burden on the healthcare system [1]. Vertebral fractures (VFs) account for about 50% of all osteoporotic fractures annually and are the most prevalent complication of osteoporosis [2, 3]. Vertebral augmentation (VA) procedures, also known as balloon kyphoplasty (BKP) or vertebroplasty, are a minimally invasive surgery for symptomatic osteoporotic vertebral compression fractures (OVCFs), which can facilitate biomechanical stability and functional recovery in the shorter term and may reduce the mortality rate compared to those treated non-surgically [4, 5]. However, the occurrence of new vertebral fractures (NVFs) within two years of VA, referred to as “imminent fractures”, was reported in about 18.4–34.8% of patients [6–8]. Additional VFs occurred sooner in VA patients than in patients with non-surgical management [9]. Moreover, the morbidity of new vertebral fractures was associated with increased mortality [10]. Predicting imminent NVFs after VA within two years is critical for patient prognosis and selection of appropriate therapy.

Magnetic resonance imaging (MRI) is the most appropriate imaging modality for evaluating patients with new OVCFs. T1-w and T2-w MRI images have distinct signal intensity patterns that can reflect pathological changes [11]. Radiomics is a non-invasive reproducible method for extracting high-throughput quantitative image features from medical diagnostic images using data characterization algorithms or statistical analysis, and it has demonstrated promising results in the diagnosis of osteoporosis and prediction of vertebral fracture occurrence [12–14]. The radiomics features derived from T1-w imaging reflecting the spatial heterogeneity of vertebral bone marrow linked to skeletal fragility has been investigated [15]. MRI is integral to the routine management of patients with NVFs, and the additional value of radiomics based on MRI for predicting the risk of NVFs after VA within two years warrants further investigation and clinical application.

The traditional COX proportional hazard (CPH) model has been frequently utilized to identify risk factors for predicting the early prognosis of patients [16, 17]. However, the approach is based on the assumption of linearity and cannot describe the nonlinear and complex relationships that may occur in biological systems, resulting in poor predictive performance [18, 19]. The random survival forest (RSF) model, a novel machine learning-based algorithm, has been shown to accurately deal with potentially nonlinear variables and censored survival data [20, 21].

This study aimed to develop and validate an MRI-based radiomics RSF model and compare the performance of the RSF model with the CPH model in predicting imminent NVFs for patients after VA.

## Methods

### Study participants

The current multi-institutional study using anonymous data was approved by the institutional review board of each participating institution, and the requirement for written informed consent was waived. A total of 235 eligible patients from the Fourth Affiliated Hospital of Guangzhou Medical University and Huizhou Central People's Hospital were enrolled. Patients treated at the Fourth Affiliated Hospital of Guangzhou Medical University between July 2013 and March 2020 were assigned in a 7:3 ratio to the training and internal validation sets, while 38 patients treated at Huizhou Central People's Hospital between October 2014 and September 2020 were assigned to the external validation set. Inclusion criteria included: i) female patients aged > 50 years and male patients aged > 60 years, ii) patients diagnosed as acute OVCFs based on the presence of bone marrow edema on preoperative spinal MRI and then received VA procedures. The exclusion criteria included: (1) patients with fractures caused by infection, tumor, or high-energy trauma; (2) patients who declined to follow-ups or died during the follow-up period.

**Fig. 1:**
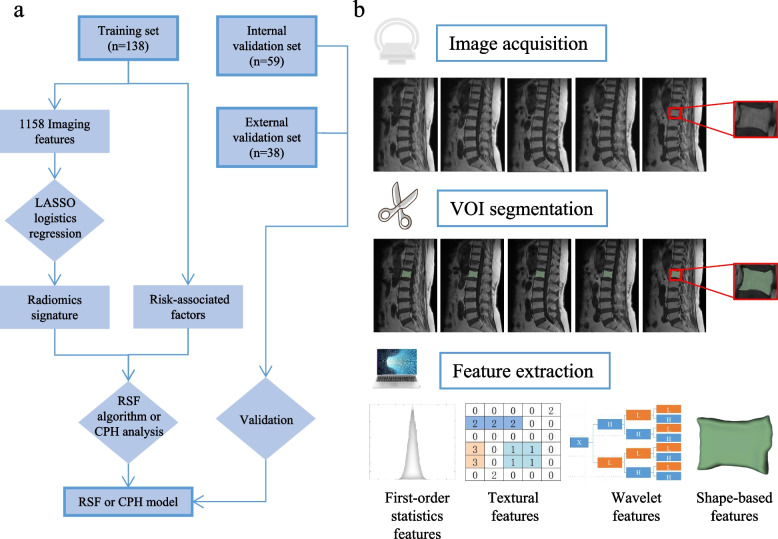
The study flowchart and the workflow of radiomics. **a** The flow chart for the three data sets in the study. **b** The 3-step radiomics workflow presents the procedure of radiomics analysis: image acquisition, volume of interest (VOI) segmentation, and radiomics feature extraction

Baseline clinical data (age and sex) and information on VA procedures (number of treated vertebrae, location of treated vertebrae, and surgical procedures) were collected from the medical records of both the hospitals. After analyzing all the MRI scans, two radiologists (radiologists 1 and 2 with 25 and 15 years of experience in musculoskeletal MRI interpretation, respectively) documented MRI findings, such as the presence of previous VF, previous multiple VFs, and intravertebral cleft (IVC). An IVC is a cavity within the vertebral body typically filled with gas or liquid [22]. The flowchart of this study is depicted in Fig. 1(a).

### Image acquisition

All enrolled patients underwent preoperative spinal MRI with a 1.5 T MR scanner within a week before surgery. The detailed MR scan protocols are presented in Supplementary Table 1. All enrolled patients were followed up every three months postoperatively until NVFs occurred or the two-year follow-up period ended, whichever came first. During the follow-up period, patients who suffered from recurrent low back pain or difficulty walking were recalled to the hospital for a spinal MRI at any time. The postoperative spinal MRI was done at the last follow-up visit to determine whether NVFs occurred. Furthermore, based on the results of the postoperative spinal MRI, the patients were divided into the NVFs and without NVFs groups.

### Volumes-of-interest segmentation and radiomics feature extraction

The regions of interest (ROIs) of the L1 vertebral body were segmented slice-by-slice using the free and open-source 3D Slicer software (Harvard Medical School, version 4.13.0) by two radiologists who were blinded to the patient’s outcomes to reduce the operator’s biases. If previous VFs existed or were treated in the L1 vertebral body, an adjacent vertebral body (T12 or L2) was chosen. Chronic fractured vertebrae, characterized by a 25% or more reduction in vertebral height but no abnormal signals in spinal MRI, were also excluded [23]. Moreover, the volumes of interest (VOIs) of selected vertebral bodies were constructed by stacking up the corresponding ROIs. Figure 1(b) depicts the radiomics workflow.

We followed a two-step procedure to account for the impact of image preprocessing methods. All images were resampled to a voxel size of 1 × 1 × 1 mm to standardize the voxel spacing, and z-score normalization was performed to validate the repeatability of the feature extraction. A total of 1158 radiomics features, including first-order features, textural features, shape-based features, and wavelet features, were extracted from every VOI using the pyradiomics platform (version 3.0.1) implanted in Python software.

### Feature selection and radiomics signature construction

The intraclass correlation coefficient (ICC) was used to determine the inter-observer variability. First, 70 vertebrae were randomly selected to evaluate the inter-observer reliability of radiomics features. Second, the radiomics characteristics with ICCs > 0.9 were considered reliable and included in the subsequent analysis. Furthermore, the Mann–Whitney U tests were performed to determine whether the two groups had statistical differences in radiomics features. The features with *p*-value > 0.05 were excluded. The optimal features subset was determined in the training set using the most extensively used least absolute shrinkage and selection operator (LASSO) regression algorithm [24]. The penalty parameter (λ) was tuned using fivefold cross-validation. A radiomics signature was developed based on the radiomics score, which was calculated for each patient using a linear combination of included features weighted by their respective LASSO coefficients. Furthermore, a stratified analysis of all enrolled patients within NVFs and without NVFs groups was also performed.

### Construction and performance assessment of the prediction models

The discriminatory power of individual variables, including radiomics signature, MRI findings, and clinical variables, was first assessed before constructing the prediction models, using time-dependent areas under the receiver operating characteristic curve (AUC). Two widely used methods were conducted in the training set to identify final risk-associated variables: RSF algorithm determined from ensemble learning of decision trees and CPH clustering analysis based on the *p*-value ranking. The selected variables were then fused into a single prediction model using the RSF algorithm or CPH analysis. Moreover, the reliable predictive performance of models was trained with fivefold cross-validation.

We used five consensus methods to assess model performance in different data sets. Model discrimination was evaluated using Harrell’s C-index and time-dependent AUC, and the dynamic time-dependent measure was measured to be two years. Furthermore, calibration curves of two models were constructed in all sets, displaying the estimated vs. actual 2-year risk probability of imminent NVFs. Decision curve analysis (DCA) was used to assess clinical usefulness, which was demonstrated by calculating the net benefits at different threshold probabilities. Overall prediction performance was evaluated using time-dependent Brier scores.

### Statistical analysis

All statistical analyses were performed using free Python software (version 3.7.1) and SPSS (version 26.0). The mean (standard deviation, SD) was used to describe normally distributed continuous variables and was compared using the Student t-test, whereas the median was used for non-normally distributed continuous variables and was compared using the Mann–Whitney U test. Categorical variables were represented by number (%). Detailed information about the LASSO logistic regression algorithm was provided in the “LassoCV” package. Models were constructed using the “COXPHSurvivalAnalysis” and “RandomSurvivalForest” modules. Statistical significance was determined by a two-sided *p*-value < 0.05.

## Results

### Patient characteristics

Table 1 summarises the details of NVFs-associated risk factors of the patients. Among all 235 patients, NVFs were present in 51.9% of patients (122 of 235). The median duration of follow-up was 17 months for the training set, 15 months for the internal validation set, and 18 months for the external validation set. The occurrence of NVFs was similar across the three data sets (*P* = 0.166, log-rank test).

**Table 1:** Patient characteristics

	Training set (*n* = 138)		Internal validation set (*n* = 59)		External validation set (*n* = 38)	
Characteristic	without NVFs	NVFs	without NVFs	NVFs	without NVFs	NVFs
Age, yr						
Mean (± SD)	70.9 (± 8.6)	75.8 (± 8.3)	72.4 (± 10.2)	75.3 (± 7.8)	74.8 (± 6.8)	78.1 (± 7.4)
Sex						
Male	16 (25.0)	14 (18.9)	7 (25.9)	9 (28.1)	4 (17.4)	3 (20.0)
Female	48 (75.0)	60 (81.1)	20 (74.1)	23 (71.9)	19 (82.6)	12 (80.0)
Surgical procedure						
VP	32 (50.0)	49 (66.2)	20 (74.1)	23 (71.9)	17 (73.9)	10 (66.7)
BKP	32 (50.0)	25 (33.8)	7 (25.9)	9 (28.1)	6 (26.1)	5 (33.3)
Number of treated vertebra(e)						
1	55 (85.9)	63 (85.1)	21 (77.8)	22 (68.8)	21 (91.3)	12 (80.0)
≥ 2	9(14.1)	11 (14.9)	6 (8.1)	10 (31.2)	2 (8.7)	3 (20.0)
Location of treated vertebra(e)						
non-TL-Junction	19 (29.7)	35 (47.3)	5 (18.5)	14 (43.8)	7 (30.4)	5 (33.3)
TL-Junction	45 (70.3)	39 (52.7)	22 (81.5)	18 (56.2)	16 (69.6)	10 (66.7)
IVC						
Negative	60 (93.8)	47 (63.5)	25 (92.6)	25 (78.1)	19 (82.6)	9 (60.0)
Positive	4(6.2)	27 (36.5)	2 (7.4)	7 (21.9)	4 (17.4)	6 (40.0)
Previous VF						
Negative	46 (71.9)	33 (44.6)	22 (81.5)	12 (37.5)	19 (82.6)	9 (60.0)
Positive	18(28.1)	41 (55.4)	5 (18.5)	20 (62.5)	4 (17.4)	6 (40.0)
Previous Multi-VF						
Negative	57 (89.1)	52 (70.3)	26 (96.3)	25 (78.1)	22 (95.7)	13 (86.7)
Positive	7 (10.9)	22 (29.7)	1 (3.7)	7 (21.9)	1 (4.3)	2 (13.3)

Except where indicated, data are numbers of patients, with percentages in parentheses

*NVF* new vertebral fracture, *VF* vertebral fracture, *VP* vertebroplasty, *BKP* Balloon Kyphoplasty, *TL-Junction* The treated vertebrae located at the level of T12-L2, *IVC* intravertebral cleft

### Feature selection and radiomics signature construction

A total of 1158 radiomics features were extracted from each VOI of the selected vertebral body on T1-w MRI images. The stability of the 1158 features was first ranked using ICCs, and 677 reliable features were then selected for subsequent analyses. Following the Mann–Whitney U test, 514 features were found to be significantly different between the NVFs and without NVFs groups. Among 514 features in the training set, ten key radiomics features with nonzero coefficients were selected using the LASSO logistic regression algorithm (Supplementary Fig. 1(a) and (b)). Finally, these ten independent radiomics features were used to generate the radiomics signature, and their corresponding coefficients are presented in Supplementary Table 2.

According to the maximum Youden index in all sets, 0.527 was selected as the optimal radiomics score cut-off value. All patients were categorized into low- or high-risk groups based on the optimal cut-off value. The waterfall plot (Supplementary Fig. 1(c)) demonstrated the distribution of radiomics scores among all enrolled patients divided into different groups, with the dividing line drawn at the cut-off value.

### Construction of the prediction models

Table 2 depicts the time-dependent AUC (2 years) of different factors. The time-dependent AUC (2 years) of the radiomics signature was 0.805 (*p* < 0.001), which was considerably higher than other variables in predicting imminent NVFs after VA. Two MRI findings, including the presence of IVC and previous vertebral fracture, and age also demonstrated a moderate predictive performance (time-dependent AUC (2 years) = 0.651, 0.636, and 0.665, respectively).

**Table 2:** The time-dependent AUC (2 years) of different factors associated with NVFs

factors	time-dependent AUC (2 years)	95% CI	*P*
Radiomics signature	0.805	0.729, 0.867	< 0.0001^a^
Age	0.665	0.580, 0.743	0.0004^a^
Sex	0.530	0.444, 0.616	0.7324
Surgical procedure	0.581	0.494, 0.664	0.0532
Number of treated vertebra(e)	0.504	0.418, 0.590	0.8944
Location of treated vertebra(e)	0.588	0.501, 0.671	0.0318^a^
IVC	0.651	0.565, 0.730	< 0.0001^a^
Previous VF	0.636	0.559, 0.726	0.0008^a^
Previous Multi-VF	0.594	0.507, 0.677	0.0046^a^

*CI* confidence interval, *IVC* intravertebral cleft, *VF* vertebral fracture, *AUC* areas under the receiver operating characteristic curve

^a^*P* < 0.05

**Fig. 2:**
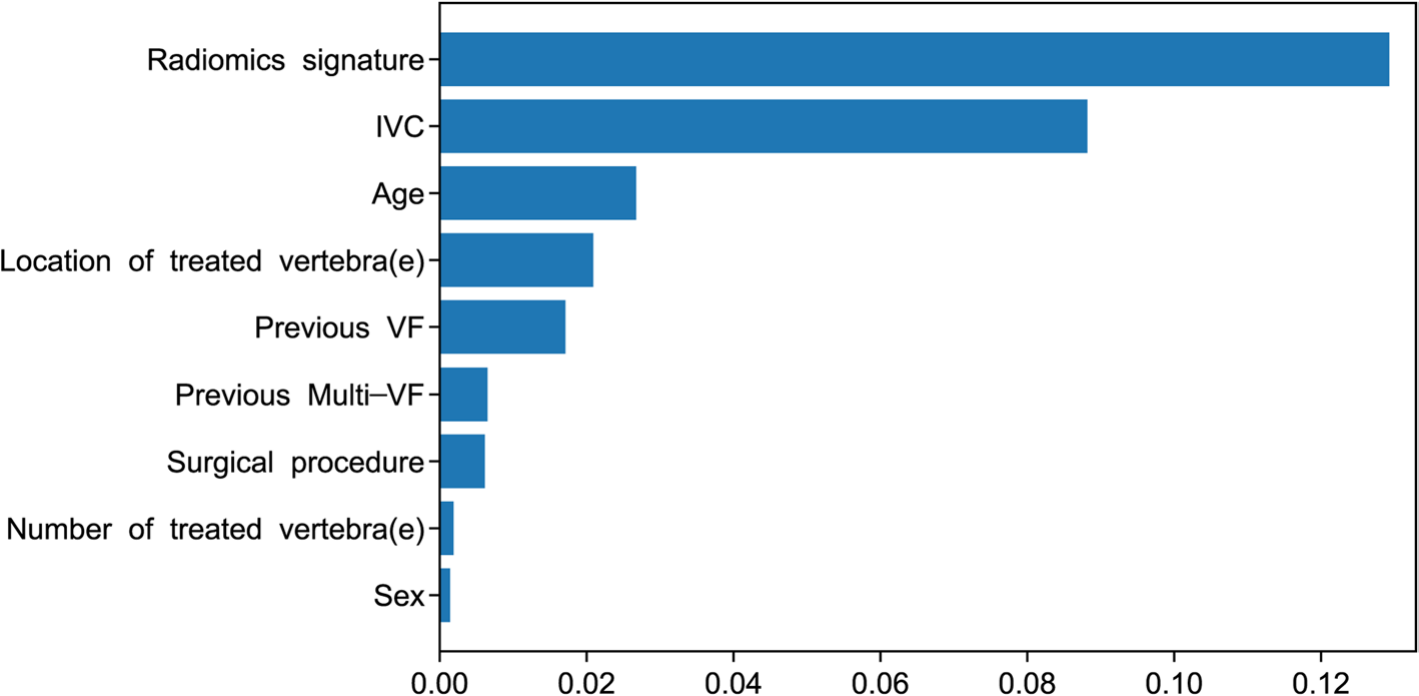
The variable importance plot based on RSF algorithm. RSF = random survival forest, IVC = intravertebral cleft, VF = vertebral fracture

The radiomics signature and the presence of IVC were the two factors most strongly correlated with the risk of imminent NVFs of the CPH analysis (Table 3). Furthermore, the two most critical variables of the RSF algorithm (Fig. 2) were the same as those of the CPH model. Radiomics signature and the presence of IVC were identified as independent risk factors in our study using the RSF algorithm or CPH analysis in the training set, and two models incorporating these two independent risk factors were constructed.

**Table 3:** Uni-variate and Multi-variable COX Analysis of NVFs-associated factors in the training set

	Uni-variate analysis		Multi-variate analysis	
factors	Hazard Ratio (95% CI)	*P*	Hazard Ratio (95% CI)	*P*
Radiomics signature	13.682 (7.678, 25.802)	< 0.001^a^	8.753 (2.746, 17.735)	0.001^a^
Age	1.040 (1.012, 1.069)	0.005^a^	1.002 (0.971, 1.034)	0.893
Sex	0.980 (0.548, 1.754)	0.946	-	-
Surgical procedure	1.534 (0.947, 2.485)	0.082	-	-
Number of treated vertebra(e)	0.780 (0.410, 1.482)	0.448	-	-
Location of treated vertebra(e)	1.631 (1.032, 2.576)	0.036^a^	1.348 (0.821, 2.212)	0.238
IVC	0.318 (0.197, 0.516)	< 0.001^a^	0.422 (0.255, 0.698)	0.001^a^
Previous VF	0.488 (0.308, 0.774)	0.002^a^	0.834 (0.453, 1.534)	0.560
Previous Multi-VF	0.498 (0.302, 0.821)	0.006^a^	1.065 (0.567, 2.000)	0.845

*CI* confidence interval, *VF* vertebral fracture, *IVC* intravertebral cleft

^a^*P* < 0.05

### Assessing and comparing models’ performance

**Fig. 3:**
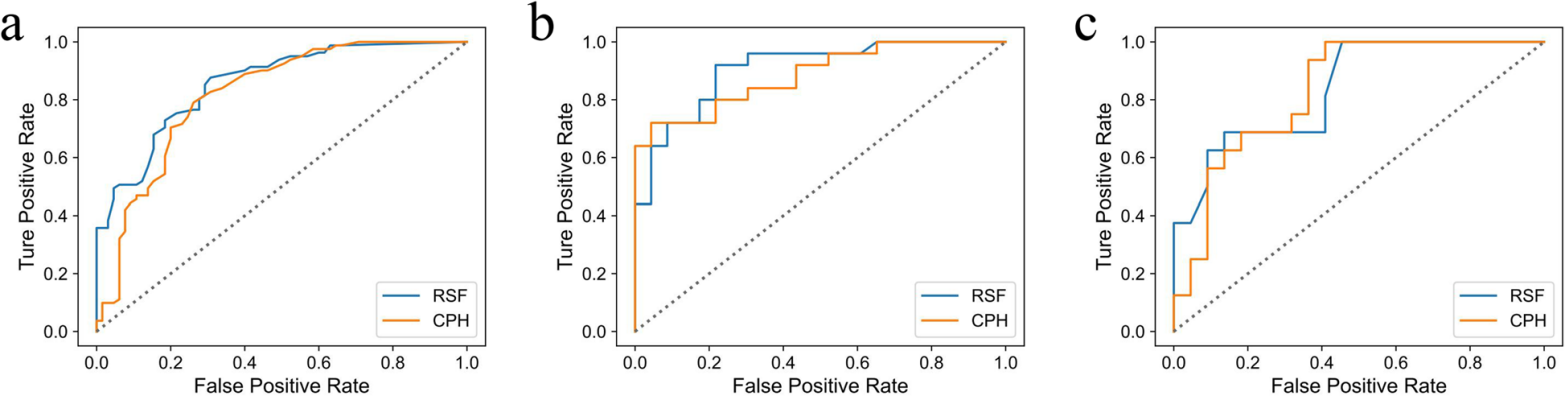
Comparison of time-dependent AUC (2 years) between the RSF model and CPH model. **a** training set, **b** internal validation set, **c** external validation set. AUC = areas under the receiver operating characteristic curve

Harrell’s C-index and time-dependent AUC (2 years) were used to compare the model discrimination. In training, internal, and external validation sets, the RSF model was found to be more discriminational with C-index of 0.763, 0.773, and 0.731, respectively, compared to the CPH model (0.711, 0.711, and 0.707, respectively) when cross-validation was performed. As shown in Fig. 3, the time-dependent AUC (2 years) of the RSF model were 0.855, 0.907, and 0.839 (*p* < 0.001 for all) in three data sets, which were greater than the CPH model (time-dependent AUC (2 years) = 0.816, 0.885, 0.832, respectively, *p* < 0.001 for all).

**Fig. 4:**
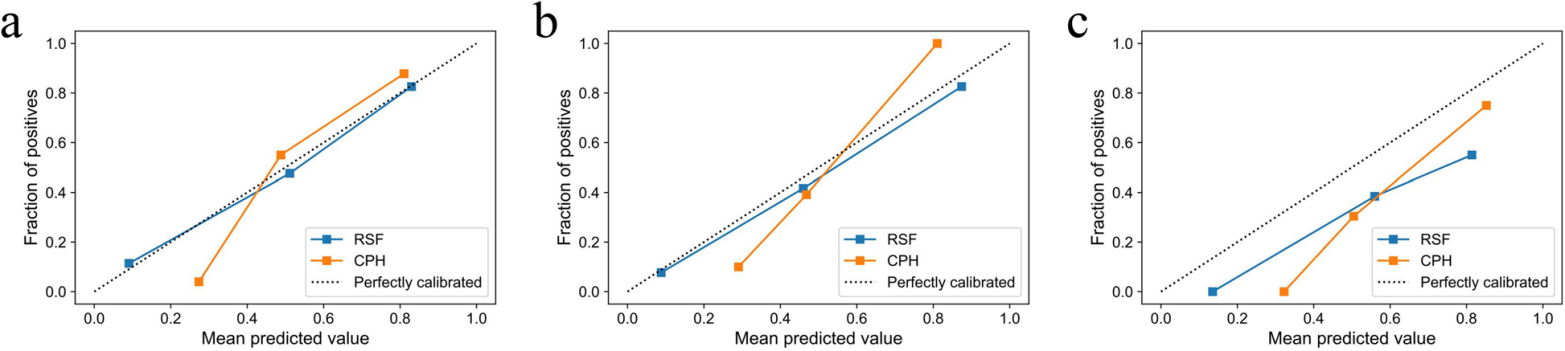
Calibration curves of two models in the training set (**a**), internal validation set (**b**) and external validation set (**c**). The x axis and y axis show the predicted probabilities and actual probabilities of having NVFs, respectively. The diagonal gray dotted line represents perfect prediction, and the solid line represents the performance of the model. The solid line has a closer fit to the dotted line, which represents a better calibration

**Fig. 5:**
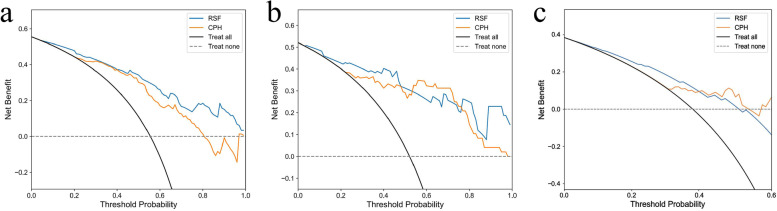
Decision curve analyses of two models in the training set (**a**), internal validation set (**b**) and external validation set (**c**). The net benefit was plotted versus the threshold probability. The black and gray lines represent the hypothesis that all patients and no patients suffered NVFs after VA, respectively

In the three data sets, the calibration curves of the RSF model showed better overall agreement with the actual outcome in the probability of imminent NVFs than the CPH model (Fig. 4). In the DCA, the RSF model demonstrated a higher net benefit over CPH model for a wide range of threshold probability in the training set, indicating its better clinical usefulness. Moreover, comparable results were observed when DCA was performed in two validation sets (Fig. 5). In terms of overall performance, the time-dependent Brier score (2 years) of the RSF model (0.156, 0.151, and 0.146, respectively) was lower than the CPH model (0.191, 0.179, and 0.156, respectively).

## Discussion

A machine learning-based risk prediction model incorporating radiomics signature and IVC was developed and validated in the current study. According to our knowledge, this is the first study to develop a machine learning model using radiomics features extracted from MRI for individualized risk prediction of NVFs after VA within two years. The RSF model is an innovative machine learning model specifically designed for risk analysis. The survival tree, which is the foundation of the RSF model, enables the modification of Gini impurities of node partitioning, resulting in improved model performance. In contrast, the CPH model relies on certain assumptions. Compared to the traditional risk prediction CPH model, the machine learning-based RSF model presented a better fit to predict individual risk of imminent NVFs after VA in terms of discrimination, calibration, and clinical usefulness.

The risk of a subsequent fracture is particularly high following an acute OVCF and wanes progressively with time [25–27]. To facilitate the individualized treatment of OVCF patients after VA procedures, identification of imminent NVFs occurrence should be performed, and effective anti-osteoporotic agents should be considered to prevent these imminent fragility fractures [28]. MRI is the gold standard imaging modality for evaluating patients with new OVCFs, which is routinely performed before VA procedures [29]. Clinicians may be able to treat and manage patients at risk more effectively if they use preoperative MRI data to predict the onset of imminent NVFs [30].

By converting medical images into mineable high-dimensional data, radiomics features have been demonstrated to reflect the intrinsic characteristics of osteoporosis and vertebral fractures [31–33]. In the present study, T1-w MRI images were analyzed to identify the most significant predictive radiomics features, and a radiomics signature was developed from these radiomics features. According to time-dependent AUC (2 years), radiomics signature exhibited the best predictive performance, providing new insights into predicting imminent NVFs after VA. Moreover, the predictive performance of the radiomics signature was found to be superior to other factors in the RSF algorithm. The CPH analysis also showed that the radiomics signature was an independent predictive factor for imminent NVFs after VA. Indeed, when patients were categorized into low- or high-risk groups according to their optimal radiomics score cut-off value (0.527) based on the radiomics signature, the high-risk group (radiomics score > 0.527) had a higher probability of experiencing NVFs, identifying 77.9% (95/122) of the patients with NVFs. This categorization enables identification of patients that may require additional treatment after VA. It was reported that the presence of IVC was an important risk factor for subsequent fracture [34, 35]. IVC was identified as an independent predictor of imminent NVFs after VA, in addition to the radiomics signature. Furthermore, the radiomics signature and the presence of IVC in our RSF model can be determined from routine MRI examinations. Therefore, the prediction model is easy to use without adding additional cost or burden to the patients.

In a recent radiomics study, radiomics features were extracted from T11-L5 segments on MRI images and fused to a radiomics signature that can predict NVFs after VA [14]. However, the shape and fracture incidence of vertebrae at different segments differ, which may affect the accuracy of model predictions when all are included in the study [36, 37]. The L1 vertebral body was primarily selected because it is included on all standard spinal MRI examinations, substantially broadening its potential applications in clinical practice. And the imaging features of L1 vertebrae has been proved that it can differentiated osteoporosis/osteopenia from normal BMD, and can predict the risk of fragility vertebral fracture [38, 39]. Our model was based only on the L1 or adjacent vertebral body (T12 or L2) segmentation, which is more convenient than the T11-L5 segmentation. The study also had the advantage of approaching progression prediction through time-to-event data sets, which allowed us to obtain precise risk estimates.

There are certain limitations in the present study. First, the external validation set was relatively small, and a larger set is required to confirm the performance of the present RSF model. Second, the radiomics signature was constructed only based on T1-w MRI images. More MRI-based studies, such as short-time inversion recovery and chemical shift sequences, are needed in the future to accrue high-level evidence for clinical application. Third, our VOIs were manually outlined, which may have resulted in observer bias and increased workload. Convenient automatic segmentation of vertebrae will be investigated in the future.

## Conclusion

In conclusion, we developed and validated a robust RSF model to predict imminent NVFs after VA. This novel tool could help clinicians with postoperative follow-up and individualized treatment.

## Supplementary Information

**Additional file 1: Supplementary Table 1.** Details of parameters for 1.5 Tesla T1-w MRI imaging protocols. **Supplementary Table 2.** Radiomics features final selected by lasso regression and the coefficient to develop the radiomics signature.

**Additional file 2: Supplementary Figure 1.** Construction and Performance of the radiomics signature. (a,b) Radiomics feature selection using least absolute shrinkage and selection operator(LASSO) logistic regression. (a) Selection of the tuning parameter (λ). (b) LASSO coefficient profiles of the 514 radiomics features. (c) Waterfall plot for the distribution of radiomics score and two groups of individual patients in all enrolled patients.

## Data Availability

The datasets used and analysed during the current study are available from the corresponding author on reasonable request. The data related to this study can be made available upon reasonable request.

## Abbreviations

OVCF: Osteoporotic vertebral compression fracture
NVF: New vertebral fracture
VA: Vertebral augmentation
RSF: Random survival forest
CPH: COX proportional hazard
VOI: Volume of interest
AUC: Area under the receiver operating characteristic curve
IVC: Intravertebral cleft
LASSO: Least absolute shrinkage and selection operator
