# Supplementary material for: Development and validation of a machine learning model to predict imminent new vertebral fractures after vertebral augmentation
Source: BMC Musculoskelet Disord. 2023 Jun 9;24:472. doi: 10.1186/s12891-023-06557-w (PMC10251538; doi:10.1186/s12891-023-06557-w)
Supplement: Supplementary file 1 — Additional file 1: Supplementary Table 1. Details of parameters for 1.5 Tesla T1-w MRI imaging protocols. Supplementary Table 2. Radiomics features final selected by lasso regression and the coefficient to develop the radiomics signature. [file 12891_2023_6557_MOESM1_ESM.docx]

***Supplementary Material***

**Development and validation of a machine learning model to predict imminent new vertebral fractures after vertebral augmentation**

**Yang Jiang^1†^, Jinhui Cai^1†^, Yurong Zeng^3^, Haoyi Ye^2^, Tingqian Yang^1^, Zhifeng Liu^2*^, Qingyu Liu^1*^**

†These authors have contributed equally to this work and share first authorship

^*^These authors have contributed equally to this work and share last authorship

**^*^ Correspondence:**

Qingyu Liu

[liu.qingyu@163.com](mailto:liu.qingyu@163.com)

Zhifeng Liu

[13798168271@163.com](mailto:liu.qingyu@163.com)

1. **Supplementary Figures and Tables**
   1. **Supplementary Figures**

**Supplementary Figure 1. Construction and Performance of the radiomics signature.** (a,b) Radiomics feature selection using least absolute shrinkage and selection operator(LASSO) logistic regression. (a) Selection of the tuning parameter (λ). (b) LASSO coefficient profiles of the 514 radiomics features. (c) Waterfall plot for the distribution of radiomics score and two groups of individual patients in all enrolled patients.

- 1. **Supplementary Tables**

**Supplementary Table 1**: Details of parameters for 1.5 Tesla T1-w MRI imaging protocols

| Parameters | the Fourth Affiliated Hospital of Guangzhou Medical University | Huizhou Central People’s Hospital |
| --- | --- | --- |
| Scanner | GE (Signa HDi) | Philips (Multiva) |
| Repetition / echo time (msec) | 450/8.2 | 384/7.4 |
| Sequence | TSE | TSE |
| Bandwidth (Hz) | 260 | 231.5 |
| Thickness(mm) | 4 | 4 |
| Gap(mm) | 0.5 | 0.8 |
| Field of view (mm) | 300 | 342 |
| Voxel size(mm) | 0.9×1.3×4.0 | 0.9×1.3×4.0 |
| Flip angle (degrees) | 180 | 90 |

**Supplementary Table 2**: Radiomics features final selected by lasso regression and the coefficient to develop the radiomics signature

| Features | Coefficient |
| --- | --- |
| log-sigma-2-0-mm-3D_firstorder_Kurtosis | 0.02114282400065759 |
| log-sigma-3-0-mm-3D_glcm_Imc1 | 0.718705787701447 |
| log-sigma-3-0-mm-3D_glcm_Imc2 | -3.3164866553510395 |
| log-sigma-3-0-mm-3D_glrlm_ShortRunLowGrayLevelEmphasis | -2.4684337724724874 |
| log-sigma-4-0-mm-3D_glcm_InverseVariance | -3.776064384418479 |
| log-sigma-5-0-mm-3D_glszm_GrayLevelNonUniformityNormalized | -1.5206950925170246 |
| wavelet-HLH_glcm_MaximumProbability | -2.6138932746338672 |
| wavelet-HHL_glcm_InverseVariance | -5.768992138656398 |
| wavelet-HHL_glrlm_RunEntropy | 0.04027520082662116 |
| wavelet-HHL_glszm_SizeZoneNonUniformityNormalized | 0.027990962009973815 |
| Intercept（*β*） | 9.271788199732192 |
