# Supplementary figures and images for: Development and validation of a machine learning model to predict imminent new vertebral fractures after vertebral augmentation
Source: BMC Musculoskelet Disord. 2023 Jun 9;24:472. doi: 10.1186/s12891-023-06557-w (PMC10251538; doi:10.1186/s12891-023-06557-w)

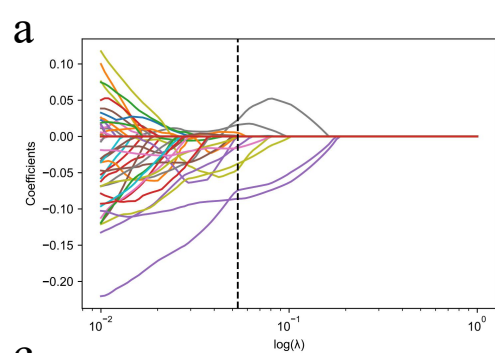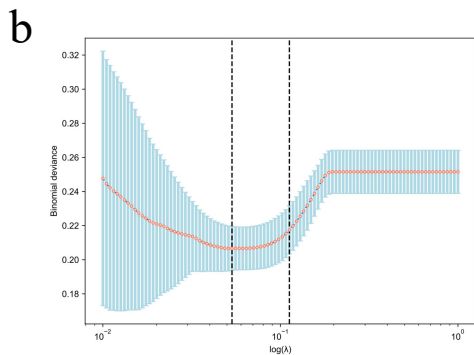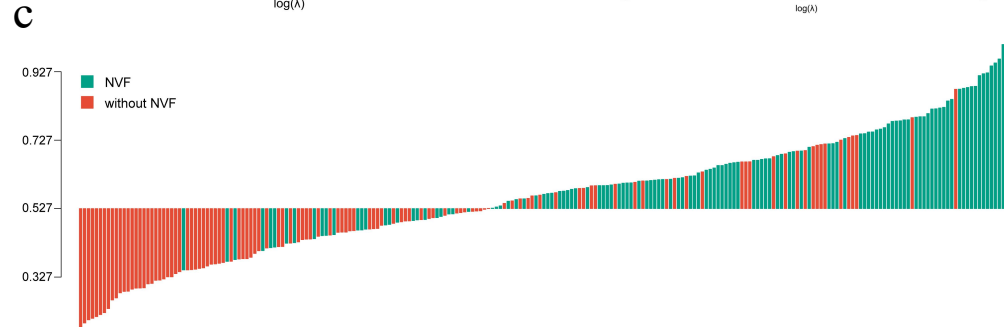

Supplement: Supplementary file 2 — Additional file 2: Supplementary Figure 1. Construction and Performance of the radiomics signature. (a,b) Radiomics feature selection using least absolute shrinkage and selection operator(LASSO) logistic regression. (a) Selection of the tuning parameter (λ). (b) LASSO coefficient profiles of the 514 radiomics features. (c) Waterfall plot for the distribution of radiomics score and two groups of individual patients in all enrolled patients. [file 12891_2023_6557_MOESM2_ESM.pdf]
